# Tropical lacustrine sediment microbial community response to an extreme El Niño event

**DOI:** 10.1038/s41598-023-33280-2

**Published:** 2023-04-27

**Authors:** Mingfei Chen, Jessica L. Conroy, Robert A. Sanford, D. Allie Wyman-Feravich, Joanne C. Chee-Sanford, Lynn M. Connor

**Affiliations:** 1 Department of Earth Science and Environmental Change, University of Illinois at Urbana-Champaign, Urbana, IL USA; 2 Department of Plant Biology, University of Illinois at Urbana-Champaign, Urbana, IL USA; 3 Department of Geosciences, The Pennsylvania State University, University Park, PA USA; 4 Department of Natural Resource and Environmental Science, University of Illinois at Urbana-Champaign, Urbana, IL USA; 5 USDA-ARS, Urbana, IL USA; 6 Present Address: Climate and Ecosystem Sciences Division, Lawrence Berkeley National Laboratory, Berkeley, CA USA

**Keywords:** Microbial ecology, Limnology

## Abstract

Salinity can influence microbial communities and related functional groups in lacustrine sediments, but few studies have examined temporal variability in salinity and associated changes in lacustrine microbial communities and functional groups. To better understand how microbial communities and functional groups respond to salinity, we examined geochemistry and functional gene amplicon sequence data collected from 13 lakes located in Kiritimati, Republic of Kiribati (2° N, 157° W) in July 2014 and June 2019, dates which bracket the very large El Niño event of 2015–2016 and a period of extremely high precipitation rates. Lake water salinity values in 2019 were significantly reduced and covaried with ecological distances between microbial samples. Specifically, phylum- and family-level results indicate that more halophilic microorganisms occurred in 2014 samples, whereas more mesohaline, marine, or halotolerant microorganisms were detected in 2019 samples. Functional Annotation of Prokaryotic Taxa (FAPROTAX) and functional gene results (*nifH*, *nrfA*, *aprA*) suggest that salinity influences the relative abundance of key functional groups (chemoheterotrophs, phototrophs, nitrogen fixers, denitrifiers, sulfate reducers), as well as the microbial diversity within functional groups. Accordingly, we conclude that microbial community and functional gene groups in the lacustrine sediments of Kiritimati show dynamic changes and adaptations to the fluctuations in salinity driven by the El Niño-Southern Oscillation.

## Introduction

In lacustrine systems, salinity is an important stress selection factor and can influence microbial diversity, community structure, and metabolic activity in surface sediments^1–5^. Lake salinity can also change abruptly, due to climate variability (e.g., from extreme precipitation events and drought), with substantial impacts to lacustrine ecosystems^6–8^. Notably, previous studies on the effects of salinity on microbial communities have focused on spatial datasets from saline and hypersaline lakes^3,9^ and estuaries with continuous salinity gradients^10,11^. Although the temporal response of microorganisms in lacustrine surface sediments to abrupt salinity changes has been documented in the literature, most studies have focused on one lake^12–14^, and few studies have examined temporal shifts in tropical lacustrine sediments.

Microbial functional groups that actively participate in element cycling also vary in response to salinity changes in lacustrine settings. As an example, the relative abundance of nitrogen fixation genes, coding an important pathway for aquatic ecosystem productivity, is often reduced with high salinity in lacustrine sediments, microbial mats, and estuarine environments^15–17^. High salinity, on the other hand, provides favorable conditions for dissimilatory nitrate reduction to ammonium (DNRA) over denitrification in estuarine sediments^18–20^. Supporting this observation, microbial communities that perform these metabolic activities also show significant changes with salinity, partly due to the extreme energetic demands of osmoregulation in high salinity conditions^1^. Thus, examining temporal changes in community composition and microbial functional groups in response to changes in salinity can improve understanding of functional group roles in element cycling under different salinity conditions in lacustrine ecosystems.

On the island of Kiritimati, Kiribati, in the central tropical Pacific, several hundreds of lakes with salinities ranging from brackish to hypersaline^21^ are an ideal natural laboratory to investigate salinity influences on microbial communities and functional groups. Here, the interannual El Niño-Southern Oscillation (ENSO) phenomenon results in significant anomalies in atmospheric moisture balance (precipitation minus evaporation)^22^. Such anomalies greatly affect groundwater, surface water area, and salinity in many of these lakes^22–24^. Thus, Kiritimati is an excellent field site to study the temporal response of microbial communities to lacustrine salinity changes. During 2015–2016, a very strong central-Pacific style El Niño led to abundant rainfall over Kiritimati^25^, increasing lake surface water area^22^, and likely leading to significant lake salinity changes. Here we investigate lake water salinity, microbial community and microbial functional groups changes in a set of lake surface (0–5 cm) sediment samples from 2019 and 2014^9^. We used samples from the same lakes taken during these two field seasons to explore the relationship between microbial community and microbial functional groups with changes in lacustrine physiochemical parameters. We hypothesize that between 2014 and 2019, the dominant microbial taxa changed from halophiles to more broadly halotolerant or mesohaline taxa, and that the relative abundance and microbial diversity of functional groups also changed as salinity decreased. We first present the physiochemical data of the lakes from 2014 to 2019 to identify environmental factors that have changed significantly between these two years and discuss the potential causes. We next compare the microbial community of 2014 and 2019 samples to determine the extent of the temporal changes in microbial community and link specific taxa showing significant changes with changes in physiochemical parameters. Finally, we analyze the relationship between salinity and dominant functional groups and the diversity within these groups using taxonomy predictions and functional gene amplicons.

## Methods

### Field description and sampling

**Figure 1:**
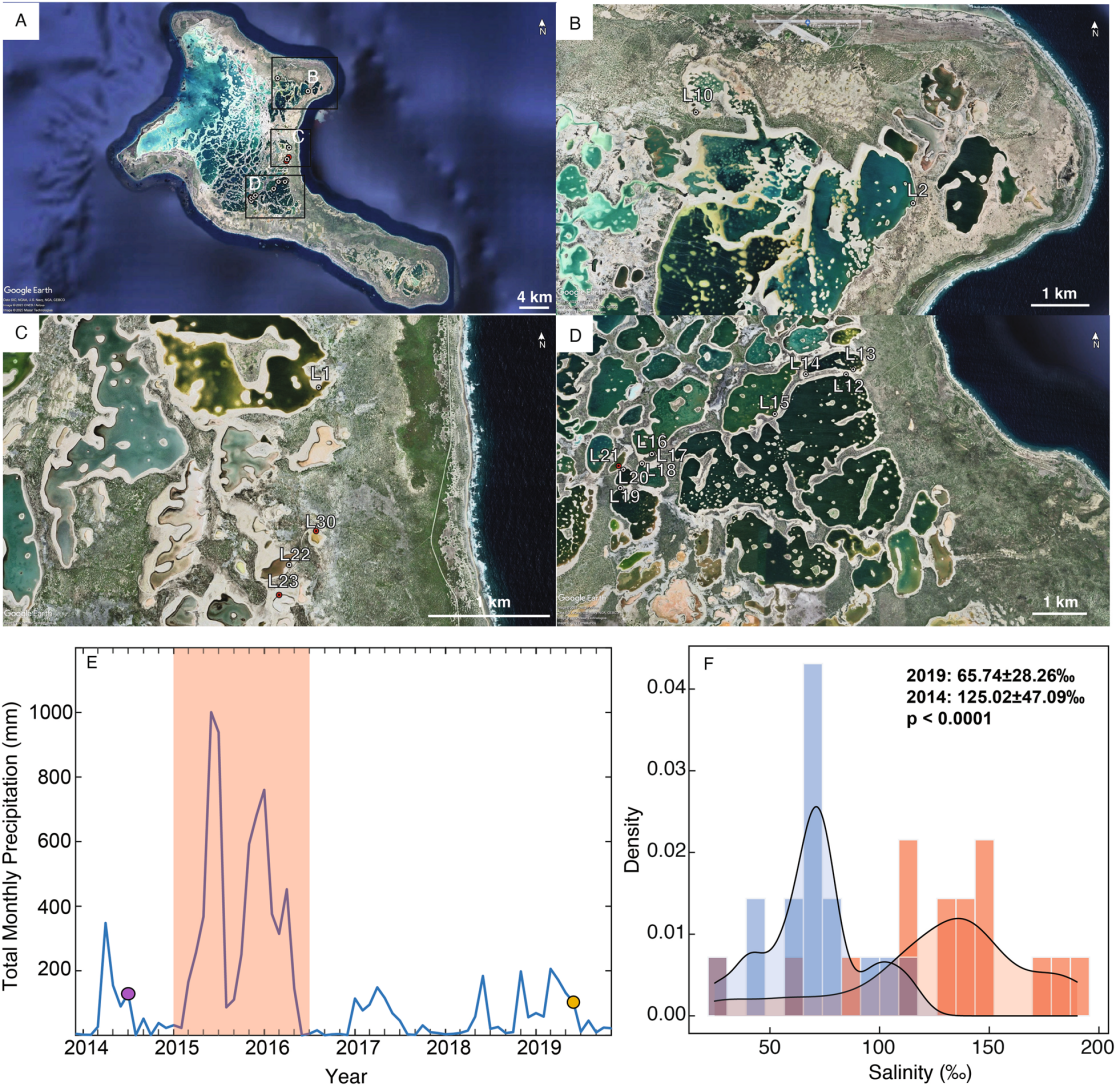
(**A**) Map of Kiritimati (Google Earth, 2021) indicating (**B**–**D**) locations of sample sites. Samples collected in both 2014 and 2019 are denoted by white circles, and samples only collected in 2019 are denoted by red circles. (**E**) Total monthly precipitation from the Kiritimati weather station. Markers indicate 2014 and 2019 field seasons. (**F**) Histogram of lake salinity variations from collected 2014 (red) and 2019 (blue) lake samples.

The Kiritimati Atoll (Republic of Kiribati, 1.9° N, 157.4° W), one of the Northern Line Island in the central tropical Pacific, is the largest coral atoll in the world with a surface area of ~ 360 km^2^. It contains hundreds of brackish to hypersaline carbonate-rich lakes, many of which are connected to a large lagoon (Fig. 1A)^21^. Nearshore sediment samples were collected from the top 5 cm of the water–sediment/microbial mat interface in the water depth of 20–50 cm from 13 lakes with salinity ranging from freshwater to hypersaline in late July and early August 2014, and June 2019 (Fig. 1A–D).

Temperature (± 0.2 °C), specific conductance relative to 25 °C (± 0.001 mS/cm), pH (± 0.2 units), and dissolved oxygen (DO) content (± 0.2 mg/L; ± 2%) were measured in situ using a YSI ProPlus multiparameter water quality sonde. Salinity was calculated in ppt from these data using the Gibbs Sea Water (GSW) Oceanographic Toolbox^26^. Alkalinity was measured on-site using a Hach Company alkalinity test kit. For cation and anion analysis, samples were double filtered by 5 μm and 0.22 μm filters and stored refrigerated in the dark in pre-acidified amber plastic bottles until analysis. The major cations concentrations were measured with ICP-OES (Perkin-Elmer, Optima 5300 DV) with a precision of ± 2%^27^. The concentration of major anions was measured with Ion Chromatography with a precision of ± 5%^28^. Water samples for water isotope analysis (δ^18^O and δ^2^H) were double filtered by 5 μm and 0.2 μm filters, collected in 30 mL brown HPDE vials without headspace and stored in a 4 ℃ freezer before analysis. The water δ^18^O and δ^2^H values were measured on a Picarro L2130‐i cavity ringdown isotopic analyzer in the UIUC Department of Geology^29^. The average precision is ± 0.1‰ for δ^18^O (VSMOW) and ± 0.8‰ for δ^2^H (VSMOW).

Sediment samples for nucleic acid extraction were sampled from 0 to 5 cm from the sediment/mat-water interface and treated with RNAlater preservative (ThermoFisher Scientific, Waltham, MA), homogenized, and stored on ice. Upon return, they were stored at − 20℃ freezer before DNA extraction and PCR amplification.

### DNA extraction and sequencing

DNA extractions for 2019 samples were performed using a modified protocol to extract DNA and RNA from soil using phenol–chloroform methods (see Supplemental Materials S1.1) in Urbana, IL. Details for DNA extractions methods for 2014 samples are given in detail in Schmitt et al. (2019). The quality and quantity of extracted DNA were determined by agarose gel electrophoresis and fluorometry (Qubit 4.0). Primer sets for bacterial- and archaeal 16S rRNA genes, N- and S-cycle genes of interest (*narG*, *nxrB*, *nirK*, *nirS*, *nosZ*, *nifH*, *amoA*, *amoB*, *nrfA*, *dsrB*, *aprA*, *soxB*) were used to generate sequence libraries (Table S1). Specific gene-targeted amplicons for downstream sequencing were generated for all samples using a Fluidigm microarray system through services available in the Functional Genomics Unit of the University of Illinois Urbana-Champaign Carver Biotechnology Center (Fluidigm Array details in Supplemental Materials S1.2). Paired-end sequencing (2 × 250 base pairs) was performed on one lane of a MiSeq Nano (v2) (San Diego, CA, USA) for 2014 samples and one lane of an Illumina NovaSeq 6000 platform (San Diego, CA, USA) for 2019 samples. Raw sequence data were deposited in MG-RAST (2014 samples, project number MGP82583) and NCBI (2019 samples, BioProject ID PRJNA76940).

### Microbial community and functional gene analyses

Following sequencing, each primer pair’s amplicon libraries from 2014 and 2019 samples were parallelly processed using Mothur^30^ and *phyloseq*^31^. For 16S rRNA genes, amplicon libraries generated from different years were examined using the exact same pipeline from *Mothur* and *phyloseq*. Briefly, paired-end reads were merged for sequences obtained from each primer pair and filtered to the expected amplicon length using Mothur^30^. For 16S rRNA genes, an OTU file based on 97% sequence similarity that included the number of OTUs found in each sample, and a taxonomy file of the consensus taxonomy for each OTU was generated from the Mothur pipeline, which was later imported into R software using *phyloseq* packages for downstream analysis. For other functional gene sequences, an OTU file based on 97% sequence similarity that included the number of OTUs found in each sample was generated from the Mothur pipeline and analyzed using *phyloseq* packages. The recovery reads from different functional genes after quality trimming are listed in Table S5.

Several methods were used to remove the potential batch effects from different primers and sequencing platforms used for 2014 and 2019 samples. First, the merged sequences for 16S rRNA from different years were aligned to the same reference sequence file (SILVA 138 database) and the same taxonomy file (see Supplementary Materials S1.3 for details). Second, for different years, the unique OTUs with the same taxonomy at the genus level were merged using tax_glom in *phyloseq* to correct for batch effects (this paper referred to as unique populations at the genus level). Genus-level comparisons were then used for downstream analysis to compare relative abundances for 16S rRNA genes in different years, and compared to another method to correct for batch effects (see Supplemental Materials S1.4 for details).

### Statistical analysis

The physiochemical parameters of lakes in 2014 and 2019 were compared and visualized using principal component analysis (PCA) to determine which parameters contribute to the variations of environmental data in the two sampling periods. In addition, to determine the parameters that changed significantly from 2014 to 2019, all parameters measured in 2014 and 2019 were assessed with a non-parametric pairwise Wilcoxon rank-sum test, and the p-values were adjusted by the Bonferroni method.

Alpha and beta diversity analyses on the genus level (PERMANOVA, multivariate permutation analysis of variance; mantel test; differential abundance) were conducted in Mothur or the R programming environment using packages *phyloseq*, *vegan*, and *edgeR*^30–33^ (Figure S1). Pairwise permutation multivariate analysis of variance (PERMANOVA) was used to determine the significance of microbial community composition differences from 2014 to 2019. In order to test how environmental parameters influence community diversity, nMDS ordination plots for bacterial and archaeal communities based on 16S rRNA gene sequences were overlaid with environmental variables (Spearman coefficients) listed in Table S2 using the function “envfit” from the *vegan* package^32^. Environmental variables that were not statistically significant were excluded from the plot and were not discussed. A Mantel test with the *vegan* package was used to assess the relationship between the microbial community and the measured environmental variables using the Bray–Curtis distance matrix.

The differences in the relative abundance of the most abundant phyla (at least > 1% in one sample) in 2014 versus 2019 lakes were assessed using the pairwise non-parametric Wilcoxon rank-sum test. This test also includes the most abundant subclasses (at least > 1% in one sample of all subclasses) of Proteobacteria due to the diversified nature of this phylum. To further explore differences in microbial communities at a finer level, a pairwise comparison of families that are more abundant in 2014 lakes compared to 2019 lakes was conducted using log twofold changes (logFC) using *edgeR*^33^, which expresses the ratio between two quantities. The correlations of relative abundance of most abundant bacterial and archaeal phyla with physiochemical parameters of all sampled lakes were visualized by “aheatmap” from package *NMF*^34^.

Functional Annotation of Prokaryotic Taxa (FAPROTAX) was used to make metabolic predictions from the valid 16S rRNA gene sequences obtained from 2014 and 2019 lake sediment samples. The predictions made in correspondence to the OTUs obtained here are based on the characterized strains with putative functional tables in the FAPROTAX database^35^. In addition, the relative abundances of different metabolic groups across the lakes and their relationship with environmental parameters were visualized by a heatmap.

The functional gene sequences acquired from 2019 lakes were input to R and processed using the same code as the 16S rRNA gene sequences. Mantel tests were used to assess the relationship between functional and taxonomic composition of the bacterial community based on Bray–Curtis distance. For the following analyses, only functional gene compositions correlated significantly with taxonomic composition were used. The alpha diversity (Shannon index) of functional genes of different lakes was calculated using the *phyloseq* package and correlated with the environmental factors of each lake. Mantel tests were also conducted to determine the correlation between functional genes and environmental factors.

## Results

### Sediment and water properties

The surface water chemistry of Kiritimati lakes varies considerably, both spatially and temporally (Table S2). Salinity in the sampled lakes ranged from 40 ppt (Lake 14, 2019) to 190 ppt (Lake 17, 2014), with a significantly higher median (137.4 ppt vs. 72 ppt) and mean value in 2014 lakes (135.2 ppt vs 74.5 ppt) (Fig. 1F). As expected, the concentrations of the most abundant cations and anions present in seawater, such as Mg^2+^, Na^+^, and Sr^2+^, are highly correlated with salinity (Figures S2, S3). Despite some differences in temperature and pH (Table S2), the median values of these parameters measured from 2014 (temperature: 30.6 °C, pH: 8, respectively) and 2019 lakes (temperature: 30.1 °C, pH: 7.99, respectively) are similar. Alkalinity, K^+^, and δ^18^O are significantly higher (*p* < 0.001 for all variables) in 2014 samples, and Ca^2+^ is significantly higher (*p* < 0.001) in 2019 samples (Table S3).

### Bacteria and archaea communities

Using 16S rRNA amplicon sequencing, we obtained 267,289 high-quality sequences generated for V4-515f primers from 2014 samples and 2,770,476 sequences for Arc519f-Bac785r from 2019 samples after filtering. There were 1163 unique genera identified for bacteria, which were assigned to 63 bacterial phyla, and 84 unique genera identified for archaea, which were assigned to 12 archaeal phyla (Figure S4). For bacterial phyla, Proteobacteria (33.02 ± 20.44%) and Bacteroidota (16.09 ± 11.16%) are dominant in most of 2014 and 2019 samples. For archaeal phyla, Thermoplasmatota and Halobacterota dominate most of 2014 and 2019 samples and Asgardarchaeota is also predominant in 2014 samples (Table 1). Nanoarchaeota shows high relative abundances in 2019 samples, however it is possible that the different PCR primers used with the 2014 samples would not have amplified sequences from this group. Other bacterial and archaeal phyla that make up at least 1% of the microbial community for at least 1 sample are listed in Table 1 and Figure S4.

**Table 1:** Median, standard deviation (1σ) and non-parametric rank-sum test (Wilcoxon test) p-values (adjusted by Bonferroni method) of the relative abundances of the most abundant bacterial and archaeal phyla (relative abundance > 1% in at least one sample, plotted in Figure S4) and Proteobacteria classes in the 2014 versus 2019 samples.

Phylum	Median relative abundance (%) 2014	Median relative abundance (%) 2019	p-value
Bacteria			
Proteobacteria:	55.03 ± 22.86	29.75 ± 6.14	**0.003**
Gammaproteobacteria	53.71 ± 24.98	9.76 ± 3.8	**0.000**
Alphaproteobacteria	2.1 ± 3.89	17.87 ± 4.83	**0.001**
Proteobacteria_unclassified	0.99 ± 0.76	0.1 ± 0.1	** < 0.001**
Bacteroidota	17.83 ± 15.25	15.02 ± 5.82	0.711
Bacteria_unclassified	11.35 ± 11.53	6.78 ± 4.89	0.080
Spirochaetota	0.63 ± 0.9	4.58 ± 2.09	** < 0.001**
Planctomycetota	0.34 ± 0.75	9.96 ± 3.78	** < 0.001**
Firmicutes	0.13 ± 10.43	0.25 ± 1.21	0.215
Verrucomicrobiota	0.13 ± 1.2	1.55 ± 0.93	0.062
Cyanobacteria	0.08 ± 0.27	5.37 ± 5.95	** < 0.001**
Desulfobacterota	0.06 ± 2.07	6.48 ± 4.82	**0.001**
Patescibacteria	0.05 ± 0.78	0.05 ± 0.06	0.839
Actinobacteriota	0.01 ± 0.04	1.27 ± 1.32	** < 0.001**
Chloroflexi	0.01 ± 0.56	1.73 ± 1.53	**0.001**
Halanaerobiaeota	0.01 ± 1.93	0 ± 0.14	0.309
Myxococcota	0 ± 0.09	2.46 ± 1.89	** < 0.001**
NB1-j	0 ± 0	1.18 ± 1.03	** < 0.001**
Zixibacteria	0 ± 0	0.63 ± 0.72	** < 0.001**
Acidobacteriota	0 ± 0.02	0.34 ± 0.96	** < 0.001**
Gemmatimonadota	0 ± 0.03	1.22 ± 1.5	** < 0.001**
SAR324_clade(Marine_group_B)	0 ± 0	0.06 ± 0.24	**0.000**
Fibrobacterota	0 ± 0.05	0.31 ± 0.52	**0.001**
Calditrichota	0 ± 0	0.25 ± 0.34	**0.000**
CK-2C2-2	0 ± 0	0 ± 0.44	0.072
Latescibacterota	0 ± 0.01	0.3 ± 0.48	**0.000**
Bdellovibrionota	0 ± 0.13	0.35 ± 0.39	**0.000**
Archaea			
Archaea_unclassified	4.08 ± 6.12	15.99 ± 10.34	0.063
Thermoplasmatota	50 ± 38.48	13.23 ± 23.76	0.151
Nanoarchaeota	0 ± 0	36.75 ± 19.34	**0.003**
Asgardarchaeota	15.68 ± 29.32	2.38 ± 3.43	0.086
Euryarchaeota	0 ± 0.02	0.02 ± 3.21	0.086
Halobacterota	0.36 ± 37.72	4.76 ± 15.34	0.906
Crenarchaeota	0 ± 12.29	3.9 ± 4.07	0.151
Aenigmarchaeota	0 ± 0	0 ± 1.37	0.086
Micrarchaeota	0 ± 0	0 ± 1.19	0.151

P-values less than 0.05 are bold.

**Figure 2:**
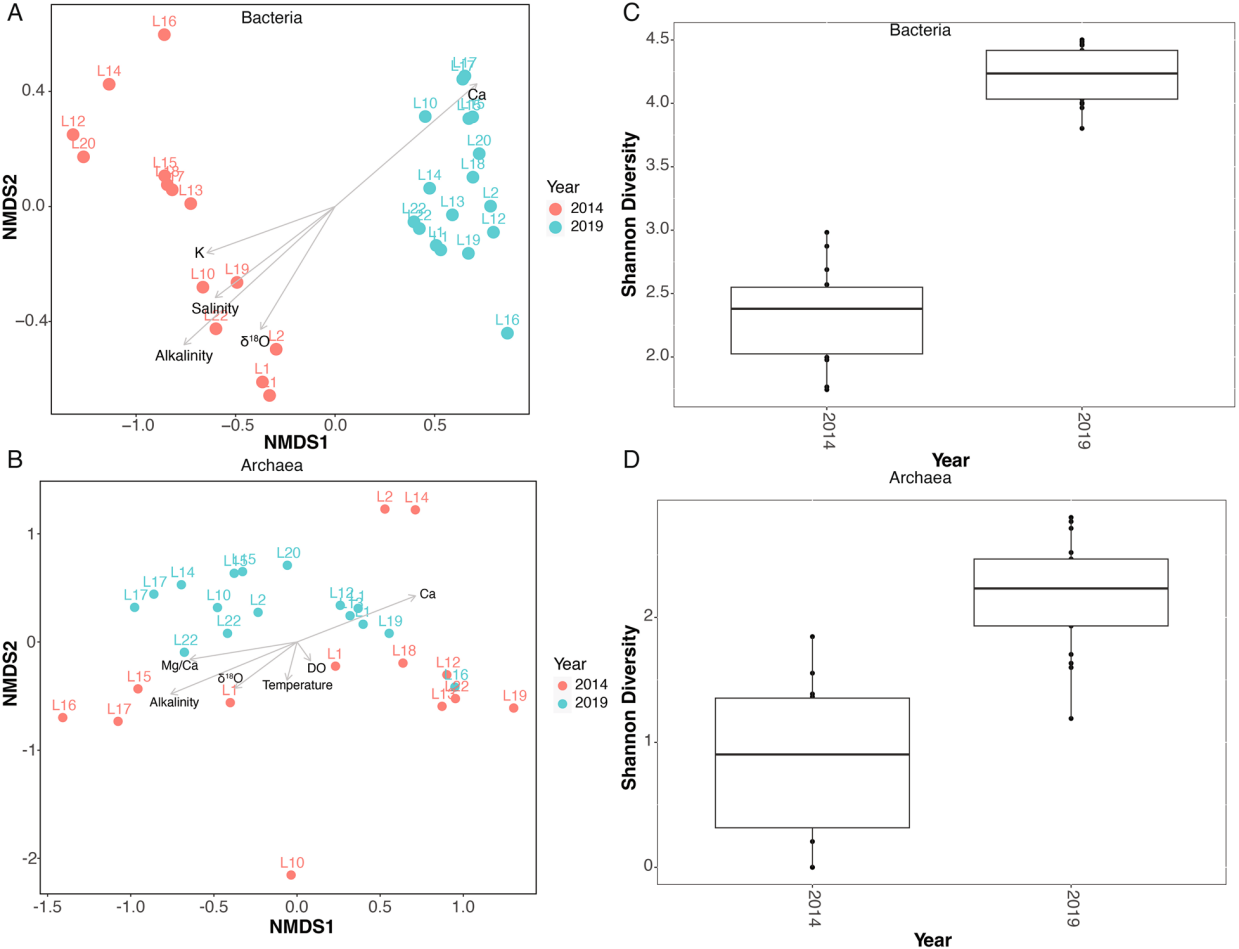
Non-metric multidimensional scaling (nMDS) and Shannon diversity plots of (**A**, **C**) bacterial and (**B**, **D**) archaeal community composition based on 16S rRNA gene sequencing data. The ordination is based on Bray–Curtis similarities overlaid with significantly correlating environmental variables (p < 0.05) plotted as vectors. Vector length is proportional to the strength of correlation between the variable and community similarity. See Table S4 for environmental fitting statistics. Samples from different years are denoted by different colors. All analyses were performed using R Statistical Software (v4.1.3; R Core Team 2022).

Bacteria Shannon diversity index values range from 1.74 to 4.50, while archaeal values range from 0 to 2.8 (Fig. 2). The sample with 0 value from the Shannon diversity index had no Archaea genera detected from one of the 2014 samples. The alpha diversity index has a significant negative correlation with salinity (Bacteria: *r* =  − 0.48, *p* < 0.01; Archaea: *r* =  − 0.54, *p* < 0.01) (Table S4). In terms of beta diversity, the pairwise PERMANOVA results indicate that both bacterial and archaeal communities are significantly different from 2014 to 2019 (Bacterial: *R*^2^ = 0.37, *p* = 0.0001, Archaeal: *R*^2^ = 0.17, *p* = 0.0001, respectively). nMDS plots also show that both bacterial and archaeal communities in the 2019 samples plot together and are distinct from the groupings found in the 2014 samples (Fig. 2). When correlating the microbial communities with physiochemical parameters, mantel test results show that the bacterial and archaeal community compositions are significantly positively correlated with salinity, alkalinity, Ca^2+^, and K^+^ (*p* < 0.05), while bacterial community composition is also significantly positively correlated with δ^18^O (Table S6).

### Functional group results

The FAPROTAX prediction analysis identified 35 metabolic functions based on 16S rRNA gene sequence data (relative abundance > 0.01%) in all sediment samples. Comparing samples from 2014 and 2019, aerobic chemoheterotrophy is significantly higher in 2014 samples and photoautotrophy is significantly higher in 2019 samples (Table S7; Fig. 5). Among the predicted microbial functions, many of them show negative correlations with salinity (*p* < 0.05), including dominant microbial functions (with median value > 1%) such as photoautotrophy and fermentation. In comparison, aerobic chemoheterotrophy is significantly positively correlated with salinity (*p* < 0.05) (Table S8).

The functional gene amplicon sequence beta diversity, done only with the 2019 samples, showed a significant correlation with the 16S rRNA community composition with the *nifH*, *nrfA*, *nirS*, *nosZ*, *aprA*, and *soxB* genes (Table S9). Salinity significantly correlates with the Shannon index of both *nifH* (*r* =  − 0.656, *p* < 0.001, *N* = 22) and *nrfA* genes (*r* =  − 0.552, *p* = 0.008, *N* = 22) encoding nitrogenase (N_2_ fixation) and nitrite reductase (NO_2_^−^ reduction to NH_4_^+^), respectively (Figure S7). Besides salinity, DO, pH, alkalinity, Sr^2+^, δ^18^O, and δ^2^H also significantly correlate with the Shannon index of one or more functional genes (Figure S7; Table 2). Salinity strongly correlates with *nifH* and *aprA* gene compositions (*r* = 0.40, 0.54; adjusted *p* = 0.002, 0.0003, respectively), along with Mg^2+^, Na^+^, Sr^2+^, Cl^−^, and SO_4_^2−^ (Table 2).

**Table 2:** Mantel test correlation results of functional genes chosen for downstream analysis with environmental factors (listed in Table S2).

Env factors	soxB			nrfA			aprA			nifH			nirS			nosZ		
	r	p	adj p	r	p	adj p	r	p	adj p	r	p	adj p	r	p	adj p	r	p	adj p
Salinity (ppt)	0.1581	0.1111	0.2698	0.4765	0.0067	**0.0228**	0.5412	0.0001	**0.0003**	0.4035	0.0006	**0.0020**	0.273	0.0035	**0.0129**	0.1631	0.0522	0.0911
Temperature (℃)	− 0.0046	0.5034	0.5744	0.0649	0.315	0.3570	0.0645	0.2809	0.3184	0.1597	0.0924	0.0982	0.0277	0.3659	0.3888	0.0506	0.3076	0.3216
DO (%)	− 0.1252	0.8075	0.8075	0.1923	0.1077	0.1831	0.1364	0.0896	0.1385	0.2669	0.0075	**0.0128**	0.0732	0.2039	0.2311	0.2444	0.0085	0.0723
pH	0.1944	0.0598	0.2292	0.2846	0.0525	0.1116	0.2945	0.0102	**0.0193**	0.3273	0.0034	**0.0083**	0.2516	0.0075	**0.0213**	0.3108	0.002	**0.0340**
Alkalinity (mg/L)	0.0294	0.3977	0.5634	0.1157	0.1852	0.2624	0.2177	0.0285	**0.0485**	0.1593	0.0588	0.0714	0.1481	0.0502	0.0948	0.1113	0.11	0.1700
Sulfide (mg/L)	0.2563	0.0161	0.2292	− 0.1432	0.8566	0.8566	0.136	0.106	0.1386	0.0291	0.3484	0.3484	0.0177	0.3944	0.3944	0.0468	0.291	0.3216
δ^13^C_DIC_ (‰)	− 0.0068	0.5128	0.5744	0.2379	0.0766	0.1447	0.1434	0.104	0.1386	0.3201	0.0051	**0.0108**	0.1626	0.0579	0.0964	0.174	0.0503	0.0911
Ca^2+^ (mg/L)	0.1858	0.0899	0.2547	0.1644	0.2081	0.2721	0.0635	0.3005	0.3193	0.2025	0.0677	0.0767	0.1565	0.0924	0.1309	0.0655	0.2821	0.3216
Mg^2+^ (mg/L)	0.147	0.1411	0.2981	0.5687	0.0035	**0.0198**	0.6422	0.0001	**0.0003**	0.4776	0.0004	**0.0020**	0.3073	0.0024	**0.0129**	0.2217	0.0294	0.0911
Na^+^ (mg/L)	0.1293	0.1578	0.2981	0.4333	0.0105	**0.0298**	0.5269	0.0001	**0.0003**	0.3776	0.0007	**0.0020**	0.2664	0.0038	**0.0129**	0.1635	0.0536	0.0911
K^+^ (mg/L)	− 0.0166	0.5406	0.5744	0.3605	0.0448	0.1088	0.5831	0.0001	**0.0003**	0.3283	0.007	**0.0128**	0.1737	0.0624	0.0964	0.1254	0.1381	0.1806
Sr^2+^ (mg/L)	0.0826	0.2488	0.3845	0.4829	0.0028	**0.0198**	0.4564	0.0003	**0.0007**	0.4135	0.0003	**0.0020**	0.2952	0.0035	**0.0129**	0.235	0.019	0.0911
Mg/Ca	0.0041	0.489	0.5744	0.1635	0.172	0.2624	0.3802	0.0036	**0.0077**	0.2323	0.0315	**0.0412**	0.1121	0.141	0.1712	0.0432	0.3216	0.3216
Cl^−^ (mg/L)	0.1922	0.0596	0.2292	0.4598	0.0059	**0.0228**	0.569	0.0001	**0.0003**	0.4101	0.0003	**0.0020**	0.2807	0.0022	**0.0129**	0.1889	0.0255	0.0911
SO_4_^2−^ (mg/L)	0.1932	0.0611	0.2292	0.4973	0.0033	**0.0198**	0.4838	0.0002	**0.0006**	0.3865	0.0005	**0.0020**	0.2466	0.0089	**0.0216**	0.1661	0.0498	0.0911
δ^18^O (‰)	0.0855	0.2163	0.3677	0.0857	0.2326	0.2824	0.0303	0.3344	0.3344	0.2207	0.0161	**0.0228**	0.108	0.1118	0.1462	0.1574	0.0452	0.0911
δD (‰)	0.1752	0.0674	0.2292	0.0553	0.3375	0.3586	0.089	0.1907	0.2316	0.273	0.0088	**0.0136**	0.2108	0.0149	**0.0317**	0.1161	0.1212	0.1717

Adjusted p-values (adjusted by Bonferroni test, shown in adj p in the table) less than 0.05 are bold.

## Discussion

### El Niño and salinity changes from 2014 to 2019

El Niño years in the central tropical Pacific have much higher precipitation rates, leading to decreased lake salinity^23,24^. The substantial salinity changes from 2014 to 2019 are likely due to the very strong central Pacific El Niño event in 2015–2016, which caused record high rainfall in the central tropical Pacific (Fig. 1E)^25^. However, individual lakes may have responded differently to this precipitation anomaly, as lakes in different areas of Kiritimati are more sensitive to precipitation, evaporation, or sea level^22^. For example, sea level can affect the water budget of lakes that are surficially connected to the main lagoon, or have a stronger subsurface connection to the ocean. Therefore, an increase in sea level during El Niño events can also result in more saline seawater flowing into such lakes, which would contradict the decline in lake salinity caused by freshwater charges from groundwater and precipitation during these periods. When lakes are isolated from the lagoon and the ocean, the major water sources are likely to be precipitation and groundwater fluxes. As a result of high precipitation during El Niño events, such lakes will experience decreased salinity. Evaporation following the termination of an El Niño event will slowly increase lake salinity, but the rates of recovery to pre-event salinity values can vary depending on the rates of the groundwater recharge. Additionally, the permeability of the lake bottoms can also differ, which can impact groundwater flow^23^. Due to the permeability of the carbonate sediment on Kiritimati atoll, there may be subterranean connections between freshwater lenses and lakes, so proximity of lakes to fresh groundwater can also affect lake salinity^21^. Moreover, bacterial mat growth provides a sealing effect and can reduce lake bottom permeability, impacting groundwater transportation^36^. Overall, the observed Kiritimati lake salinity changes are primarily attributed to the extreme El Niño event from 2015 to 2016, with variations in individual lakes due to differences in proximity to freshwater lenses, permeability, and groundwater flow.

### Community composition changes from 2014 to 2019

We found significant differences in the sediment bacterial and archaeal communities between 2014 and 2019, with these differences associated with changes in the physiochemical parameters of salinity (Tables S3 and S4; Fig. 2). Among the physiochemical parameters that changed substantially from 2014 to 2019, salinity is likely the major driver of the observed changes in microbial communities. Salinity is often considered a primary control on the composition of microbial communities in lakes, in both the water column and the underlying sediments^3,4,37–39^. High salinity can decrease the taxonomic diversity of sediment microbial communities, since high osmotic pressure during high salinity requires specific strategies for adaptation^40,41^. In addition, low salt conditions can also be deleterious for halophiles that use a “salt-in” strategy (accumulating KCl equal to NaCl within their cells), since many of the halophilic proteins of such halophiles will become unstable or denature in low salinity^42^. Therefore, the decrease in salinity and other solutes from 2014 to 2019 likely impacted the diversity of the sediment microbial communities.

**Figure 3:**
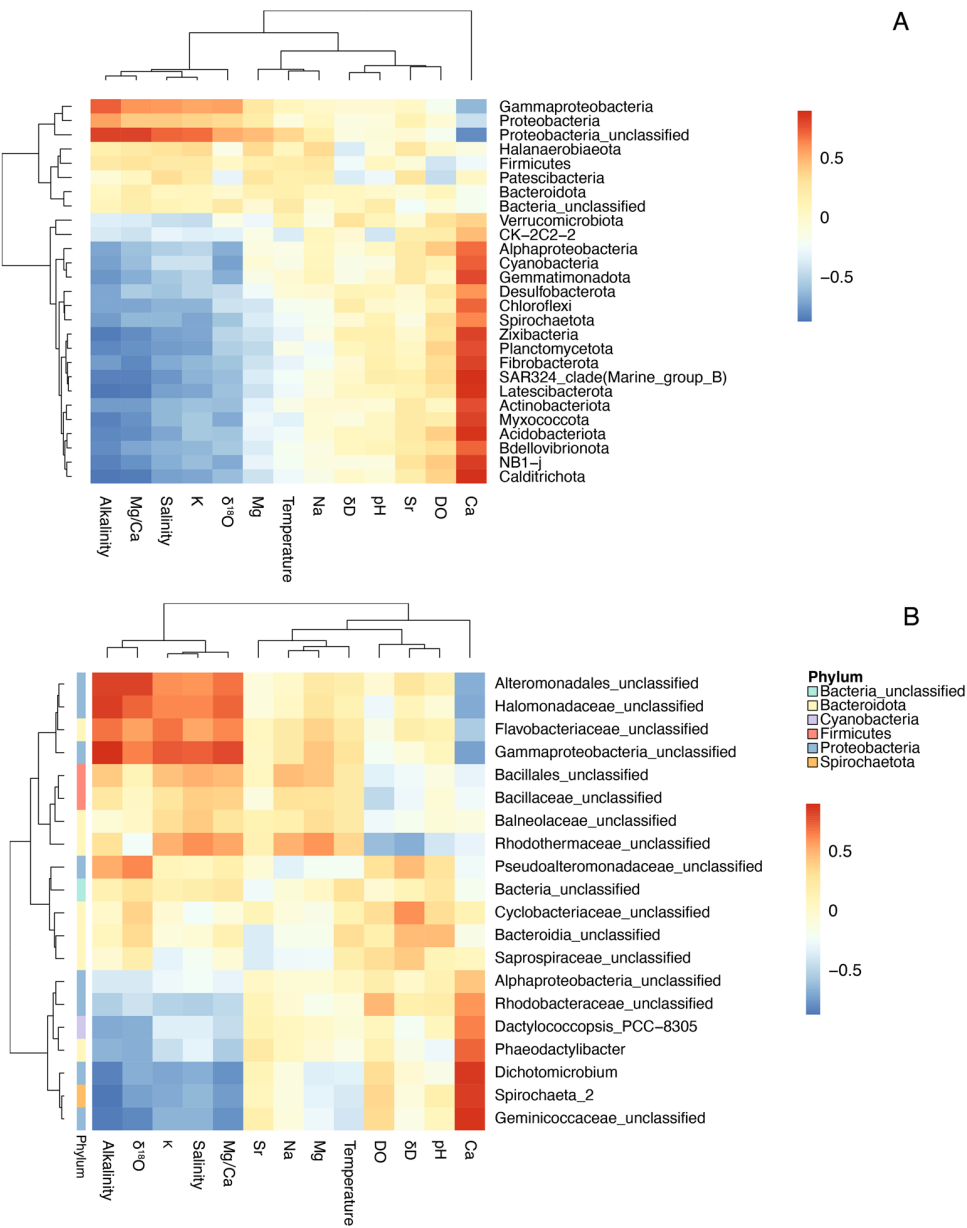
Heatmap of correlations between environmental factors and (**A**) bacterial phyla and (**B**) top 20 unique genus groups. Corresponding phylum of each genus group is also indicated in (**B**). For rows (microbial communities) and columns (environmental factors), dendrograms from hierarchical clustering using the distance and clustering methods distfun and hclustfun are made and shown in the figure. All analyses were performed using R Statistical Software (v4.1.3; R Core Team 2022, https://www.R-project.org/).

**Figure 4:**
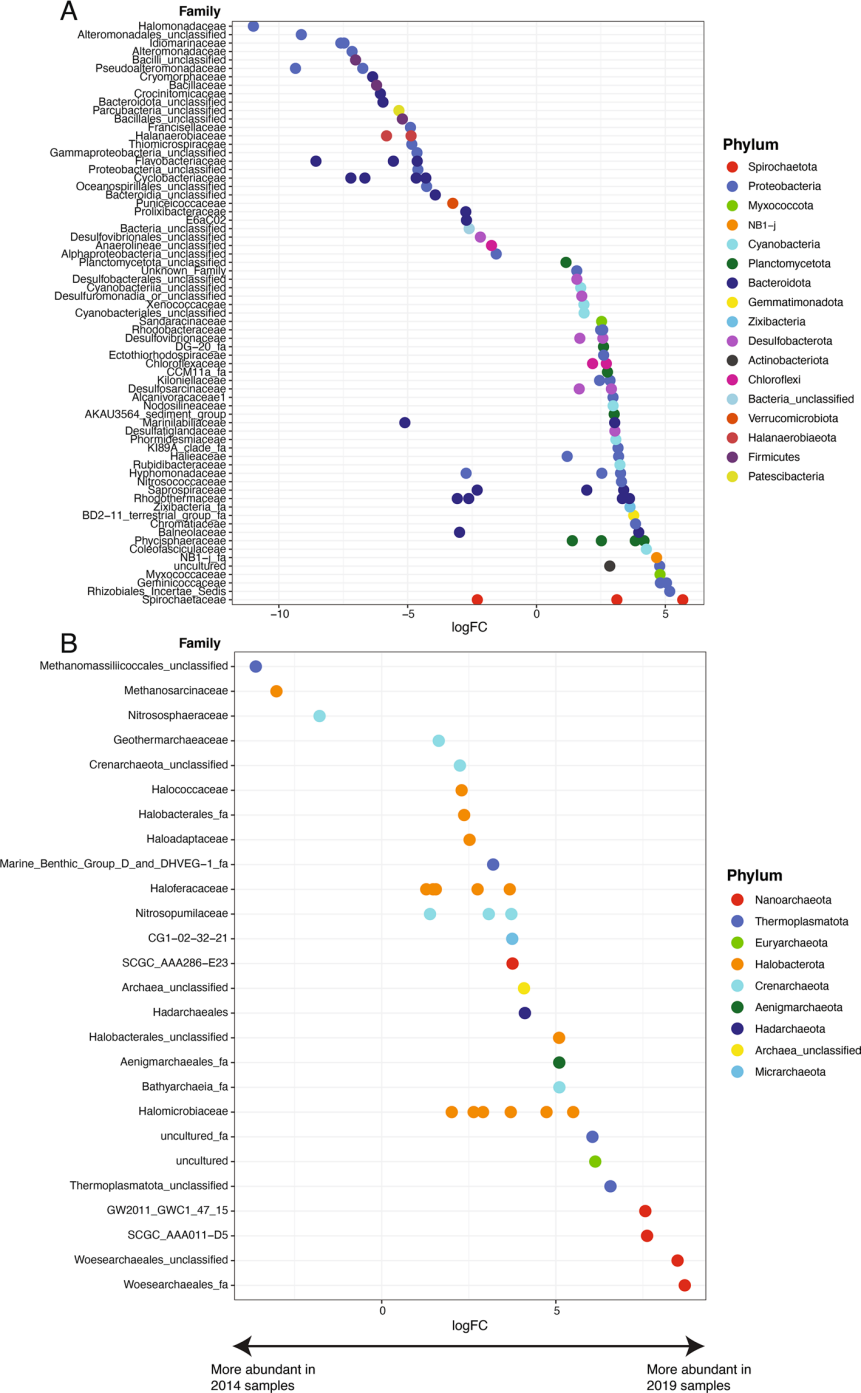
LogFC pairwise comparison between (**A**) bacterial and (**B**) archaeal families within 2014 and 2019 samples with significant differences (p < 0.05). Positive logFC values on the x-axis indicate families that are more abundant in 2019 lake sediment samples, and negative logFC values on the x-axis indicate families that are more abundant in 2014 lake sediment samples. Colors of circles denote the phyla for each listed family, shown in the legend. All analyses were performed using R Statistical Software (v4.1.3; R Core Team 2022, https://www.R-project.org/).

Considering relative abundance changes of specific phyla, classes, and families from 2014 to 2019, Gammaproteobacteria (phylum Proteobacteria) is significantly more abundant in 2014 samples (Table 1), and its abundance is positively correlated with salinity (Fig. 3A). Gammaproteobacteria include families that contain halophilic or marine bacteria that can survive in saline environments, such as Alteromonadaceae, Pseudoalteromonas, Idiomarina, and Halomonadaceae^43,44^. These families are significantly more abundant in 2014 lakes with overall higher salinities (Fig. 4). The more abundant phyla in 2019 lake sediments exhibit a significant negative correlation with salinity (Fig. 3A) and include Cyanobacteria, Desulfobacterota (formerly Deltaproteobacteria), Chloroflexi, Gemmatimonadetes, and Actinobacteria. These phyla are also more abundant in brackish lakes from the 2014 spatial survey^9^. Cyanobacteria, Chloroflexi, and Actinobacteria are generally more abundant in low-salinity settings, since salinity is an important abiotic stress for these phyla^2,45,46^. For the class Alphaproteobacteria, the families Hyphomonadaceae, Kiloniellales, Rhizobiales, Rhodobacteraceae, and Geminicoccaceae are more abundant in 2019 samples. Among them, Hyphomonadaceae and Kiloniellales are predominantly mesophilic, marine bacteria^47,48^, and Rhodobacteraceae is one of the most widely distributed bacterial lineages in marine habitats^49^. Their normal presence in marine habitats can possibly explain their high abundance in 2019 samples with lower salinity.

Additionally, some substantial changes can occur at the taxonomic level of families even if the corresponding phylum (class) shows no overall significant change from 2014 to 2019. For example, the family Cyclobacteriaceae from phylum Bacteroidota, which contains species isolated from foreshore soils and saline lakes^50,51^, has a higher relative abundance in 2014. Similarly, the families Bacillaceae and Halanaerobiaceae from phylum Firmicutes are relatively more abundant in 2014 lakes compared to 2019 lakes (Fig. 4). The Halanaerobiaceae and Bacillaceae families can either be halotolerant (family Halanaerobiaceae) or capable of forming endospores, which can help them survive in hypersaline environments^52,53^. In addition, Bacillaceae is one of the most abundant families in 2014 and shows a significant correlation with salinity (Fig. 3B). Lastly, even though class Gammaproteobacteria is more abundant in 2014 samples, the Chromatiaceae family (also known as phototrophic purple sulfur bacteria) from this class is more predominant in most of 2019 samples.

The only archaeal phylum that differs significantly between 2014 and 2019 lakes is Nanoarchaeota (Table 1), which is not present in any of the 2014 samples. As the universal primer pair used in 2014 samples^54^ shows poorer coverage of archaeal communities than the primer set used in 2019 samples^55^, we cannot exclude the possibility that Nanoarchaeota may have been present in 2014 samples but were not detected due to methodological limitations. The logFC plot of the comparison of changes in archaeal community families indicates that, surprisingly, many family members from phylum Halobacterota have significantly higher relative abundances in 2019 lake sediments (Fig. 4B). Despite this, since the detections of archaeal communities in 2014 primers were poor, comparisons of archaeal communities from 2014 to 2019 samples may not be statistically valid. When examining the 2019 spatial samples, many of the detected genera from phylum Halobacterota are only present in a small set of samples (< 5 samples) (Table S10). When looking at the 5 genera that are found in at least half of the samples (> 11 samples), 2 of them (unclassified Halomicrobiacea and uncultured Haloferacaceae) show significant positive correlation with salinity (*r* = 0.471, 0.584; *p* = 0.03, 0.005, respectively), and are among the dominant genera of the Halobacterota phylum (Table S10). To be noticed, there are dominant genera from the Halobacterota phylum that show no significant correlations with salinity. Although members from phylum Halobacterota mainly inhabit high salinity environments^56^, 16S rRNA gene results show that members are also found from low- to moderate-salinity systems^57–59^. Recent studies have shown that Halobacteria can adapt to different salinity conditions through changing membrane permeability to different solutes^60^, and can rapidly repopulate sediments when salinity drops, such as after rainfall^61,62^. Specifically, one dominant genera (*Halomarina*) that occurs in 15 lake samples can grow in a wide range of salt concentrations, and survive at low salt concentrations and can recover after prolonged exposure to distilled water^63^. Therefore, many halophilic archaea in Kiritimati lake systems may be able to adapt to abrupt changes in salinity and may not be restricted to hypersaline environments. Future isolation and characterization of those Halobacterota strains can be used to test our hypothesis.

Overall, bacterial communities show more halophilic taxa in 2014 samples and more halotolerant or marine taxa in 2019 samples, suggesting salinity was a major driver of the observed bacterial community changes. However, the diversity and changes of archaeal groups in Kiritimati lake sediments need to be further evaluated due to the limitations of the universal primer chosen in 2014 and their overall unstudied nature.

### Functional groups change with salinity

**Figure 5:**
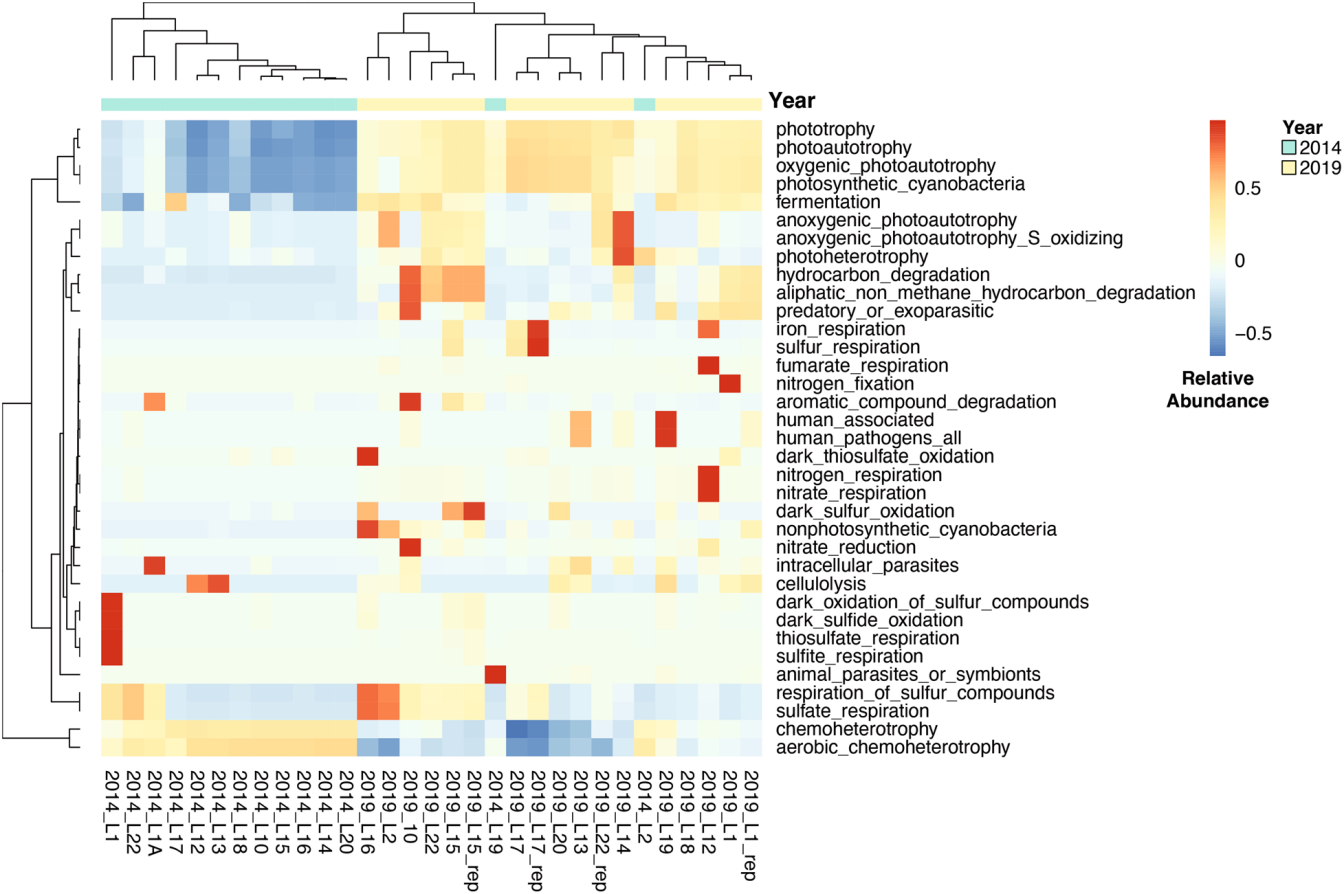
Heatmap showing relative abundances of metabolisms retrieved from Functional Annotation of Prokaryotic Taxa (FAPROTAX) for each sample. For each metabolism (rows), the relative abundances of each sample are centered, standardized and scaled to [− 1, 1] interval. For rows (metabolisms) and columns (lake samples), dendrograms from hierarchical clustering using the distance and clustering methods distfun and hclustfun are made and shown in the figure. All analyses were performed using R Statistical Software (v4.1.3; R Core Team 2022, https://www.R-project.org/).

As salinity influences the microbial community composition, the relative distribution of microbial metabolisms that directly impact lake ecosystem functions can also vary^2^. Accordingly, we modeled the relative abundances of different metabolic pathways for each sample in 2014 and 2019 using FAPROTAX, a program that uses 16S rRNA gene phylogeny (Fig. 5). The results suggest that chemoheterotrophy (48.4 ± 9.76%) is dominant in 2014 samples, while chemoheterotrophy (19.21 ± 6.89%) and phototrophy (14.12 ± 4.68%) are both important metabolisms in 2019 samples (Table S7). Furthermore, we find significant positive correlations between aerobic chemoheterotrophy and salinity (*r* = 0.49, *p* = 0.00, *N* = 31), and negative correlations between photoautotrophy and salinity (*r* =  − 0.41, *p* = 0.02, *N* = 31). Salinity can alter microbial community composition from primarily oxygenic phototrophs to primarily heterotrophs in sabkha and ocean ecosystems^64,65^. Furthermore, heterotrophs in the sediments can also utilize the organic matter substrates produced by phototrophs. For example, Gammaproteobacteria, a dominant class of heterotrophs in 2014 samples (median 53.7%), can play important roles in organic matter degradation in sediments by assimilating intermediate products (acetate) or initializing the decomposition of algal-derived organic matter^66,67^. In applying FAPROTAX to our data, the principal limitation is that FAPROTAX implicitly assumes that if all cultured members of a taxon (genus or species) can perform a particular function, then all members of the taxon (cultured and uncultured) can perform that function^35,68^. As more organisms are cultured in the future, it might lead to false generalizations. Therefore, we need to take precautions when interpreting the metabolic results obtained from FAPROTAX. Previous validation of FAPROTAX shows that some functional groups show good agreement in FAPROTAX and metagenomic results, including aerobic chemoheterotrophs and photoautothrophs^35^, which are also major functional groups shifts observed from 2014 to 2019 samples (Table S7; Fig. 5). Accordingly, FAPROTAX results may still be valid when it comes to predicting and comparing changes in aerobic chemoheterotrophy and photoautotrophy between 2014 and 2019 (Table S7; Fig. 5). In addition, previous biomarker results at Lake 30 support that microbial functional groups change with salinity over long timescales, since the absence of phytol (produced by photosynthetic organisms) and low δ^2^H values of C_16:0_ and C_18:0_ (indicating chemoautotrophic organisms) coincide with periods of high salinity over the last millennium^69^.

**Figure 6:**
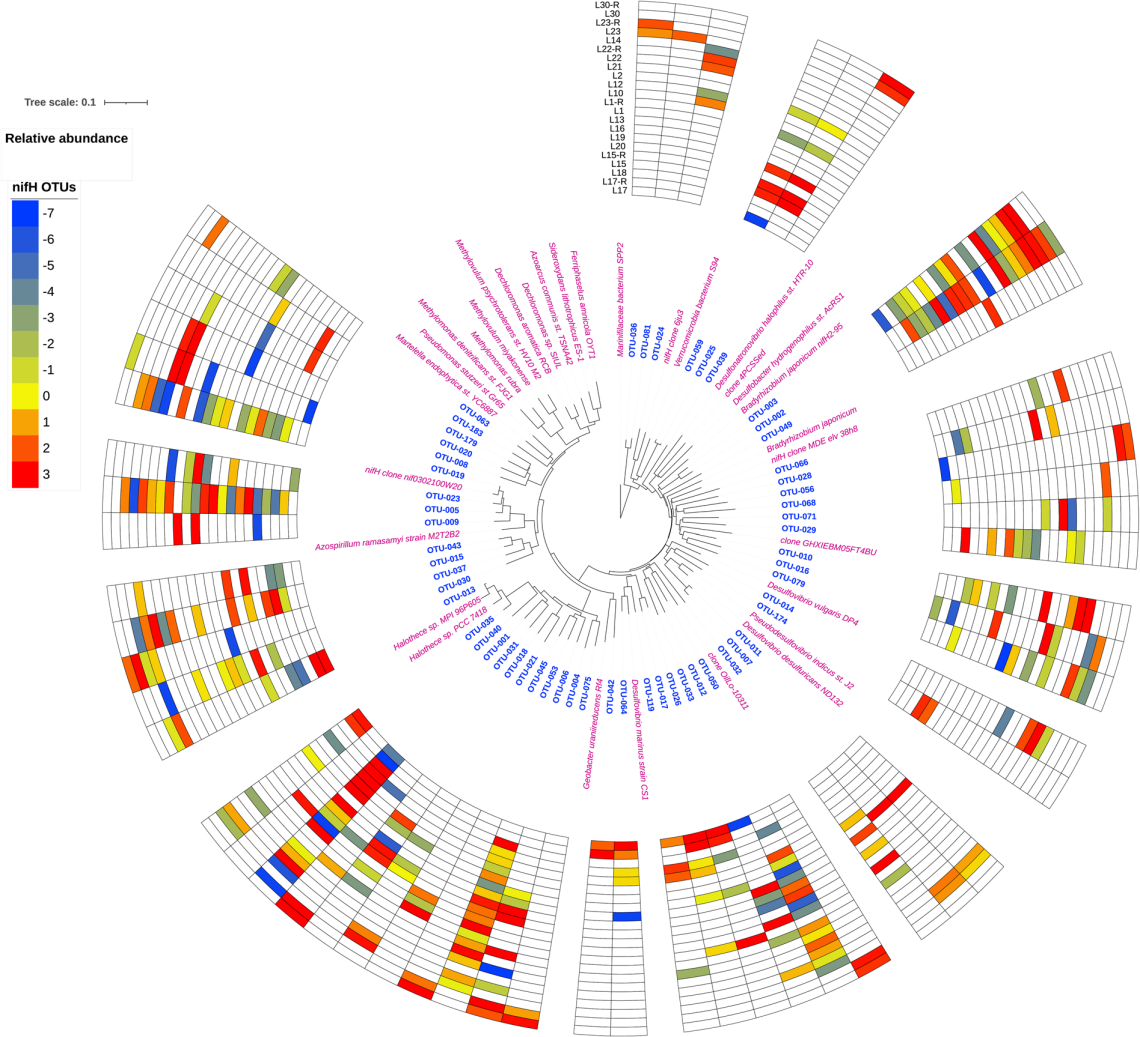
Distribition of *nifH* OTUs in the Kiritimati sediments under investigation and the phylogenetic tree for the *nifH* genes. The phylotypes under consideration (blue) comprised of the top 5 abundant OTUs identified in all the samples. The relative abundances of OTUs are presenting using a log2 transformation. The reference *nifH* genes (pink) were identified via BLAST against the NCBI-non redundant database and the gene sequences for the cultivated species were preferentially selected. The phylogenetic tree is generated using iTOL (v6, https://itol.embl.de/).

Considering the limitations of the metabolic results predicted by FAPROTAX, we also checked the functional gene results to further evaluate how salinity may impact the functional potentials. The functional gene results acquired from the 2019 spatial survey also suggest that salinity may affect microbial diversity within functional groups. As an example, the richness and diversity of *nrfA* and *nifH* genes show a significant negative correlation with salinity (Figure S7), indicating that the diversity of these genes may decrease with salinity. Additionally, salinity significantly correlated with *nifH* and *aprA* in mantel tests, showing that a change in these genes would follow salinity (Table 2). Salinity is known to increase the activity of nitrogenase (NifH) genes and decrease the abundance of Cyanobacteria in intertidal microbial mats^15^. As salinity changes, we may see changes in dominant taxa harboring *nifH* genes. According to the *nifH* phylogenetic tree results, the dominant *nifH* OTUs of hypersaline lake site 17 (113 ppt) cluster near references *Desulfovibrio marinus* and *Halothece* (Fig. 6), which are both salt-tolerant halophilic taxa^70,71^. In contrast, the predominant *nifH* OTUs of brackish Lake 30 (1.7 ppt, Fig. 6) are from taxa *Desulfobacter* that are common in marine and non-halophilic aquatic environments^72,73^. Similarly, a study conducted in Tirez Lagoon suggests that *aprA* genes can be used as indicators of salinity^74^. The relative abundances of *aprA* OTUs in lake site 17 also differ significantly from those in Lake 30 on Kiritimati (Figure S8).

Metagenomic data from a specific hypersaline lake (Lake 1) sampled in 2017^75^ provide additional information regarding how functional genes may change with salinity. The annotated *nifH* genes from metagenomic data (metagenome-assembly genomes and assembled sequences) of Lake 1 from 2017 (salinity: 44.7 ppt) cluster with *nifH* OTUs from amplicon sequences from 2019 (salinity: 71 ppt) (Figure S9), suggesting that those *nifH* genes from different years have close phylogenetic affiliations. Many of the annotated *nifH* genes from 2017 and 2019 belong to a clade of sulfate-reducing bacteria with known references that live at lower salinity conditions, including marine or slightly halophilic environments (e.g., *Desulfovibrio marinus strain CS1*, *Desulfosarcina widdelii PP31*, and *Chloroherpeton thalassium*)^70,76,77^, which is consistent with the relatively low salinity of the lakes in those two sampling years. Some of the annotated *nifH* genes from both 2017 and 2019 samples clustered with known references of halophilic Cyanobacteria (e.g., *Halothece sp. PCC 7418*). Even though halophilic Cyanobacteria from the clade “Halothece” often have optimal growth in hypersaline conditions, they may still grow suboptimally in conditions of relatively lower salinity^71^. Considering this, their presence in sediments with lower salinity suggests these Cyanobacteria can survive a wide range of salinity fluctuations in responses to precipitation anomalies on Kiritimati.

In conclusion, FAPROTAX, functional gene amplicon sequences, and previous metagenomic data altogether suggest that functional group relative abundance, diversity, and affiliated dominant taxa likely change in response to abrupt salinity changes. Also, some functional groups (e.g. Cyanobacteria) can survive under unfavorable conditions and flourish when the environment becomes more ideal in relation to ENSO events. Future metagenomic, metatranscriptomic, and isolation studies from different lakes in different years can provide additional details of how functional groups can change with salinity in these tropical sediments.

## Conclusion

In a suite of lakes with varying salinities on Kiritimati, lake salinity decreased between 2014 and 2019 due to anomalously high precipitation rates during the 2015–2016 El Niño event. Both alpha and beta diversity metrics suggest that microbial community also changed significantly from 2014 to 2019, and that these changes were correlated with salinity. Phylum- and family-level results indicate that halophilic microorganisms are more abundant in 2014 samples, whereas halo-tolerant or mesohaline microorganisms are more abundant in 2019 samples. The FAPROTAX and functional gene amplicon sequencing results suggest that the salinity-induced microbial community changes altered the relative abundance of functional groups (chemoheterotrophs, phototrophs, nitrogen fixation, denitrification, sulfate reduction) as well as microbial diversity (alpha diversity and dominant taxa) within these groups. Although some differences in primer sets and sequencing techniques could potentially account for some of these variations, we conducted thorough adjustments to account for batch effects, and our results demonstrate that these effects had negligible or no impact on our conclusions. Based on the results of our analysis and observations, we conclude that the significant decrease in salinity altered microbial communities in Kiritimati lake sediments, with a change from a dominance of halophilic microbes to mesosaline and salinity-sensitive microbes. In addition, the functional groups also changed from aerobic chemoheterotroph dominance to photoautotroph dominance in response to salinity changes. Additionally, we discovered microbial communities and functional groups living at salinity levels that were outside of their optimal growth ranges, suggesting that microbial communities on Kiritimati Island may be able to adapt to large salinity fluctuations associated with interannual precipitation anomalies. The study demonstrates how abrupt changes in salinity, induced by climate variability, can impact microbial community metabolism in tropical near-marine lacustrine environments, offering insights on the factors influencing community diversity and stability in extreme ecosystems. Moreover, this study sheds light on microbial responses to future potential climatic and anthropogenic salinity fluctuations in lacustrine environments, as previous studies showed that artificial and anthropogenic salinization and desalinization processes can substantially shape microbial communities and functions in lake systems^39,78–80^.

## Supplementary Information

Supplementary Information.

Supplementary Tables.

## Data Availability

Raw sequence data from this study has been deposited in MG-RAST (2014 samples, project number MGP82583) and NCBI (2019 samples, BioProject ID PRJNA76940). Raw sequence data for 2019 samples will be made publicly available to download upon publication. Reviewers can use the link listed below to get access to the submission of 2019 samples. https://dataview.ncbi.nlm.nih.gov/object/PRJNA769403?reviewer=jc7aunebjhi97a77ajnc7c730f.
