# Supplementary material for: Tropical lacustrine sediment microbial community response to an extreme El Niño event
Source: Sci Rep. 2023 Apr 27;13:6868. doi: 10.1038/s41598-023-33280-2 (PMC10140070; doi:10.1038/s41598-023-33280-2)
Supplement: Supplementary file 1 — Supplementary Information. [file 41598_2023_33280_MOESM1_ESM.docx]

**Tropical lacustrine sediment microbial community response to an extreme El Niño event**

Supplementary Materials

Mingfei Chen^a,1,*^, Jessica L. Conroy^a,b^, Robert A. Sanford^a^, D. Allie Wyman^c^, Joanne C. Chee-Sanford^d,e^, Lynn M. Connor^d,e^

^a^Department of Geology, University of Illinois at Urbana-Champaign, Urbana, Illinois

^b^Department of Plant Biology, University of Illinois at Urbana-Champaign, Urbana, Illinois

^c^Department of Geosciences, The Pennsylvania State University, University Park, PA, United States

^d^Department of Natural Resource and Environmental Science, University of Illinois at Urbana-Champaign, Urbana, Illinois

^e^USDA-ARS, Urbana, Illinois

^1^Current affiliation: Climate and Ecosystem Sciences Division, Lawrence Berkeley National Laboratory, Berkeley, California

*Corresponding author

Mingfei Chen

mingfeichen@lbl.gov

**S1. Supplemental materials and methods**

S1.1 *Simultaneous extraction of DNA and RNA*

Protocol for extraction of DNA and RNA from soil [1, 2]

1. Thaw frozen preserved samples (0.5 g sediment in RNAlater) and centrifuge 5 minutes at 2500xg (~4800 rpm on Eppendorf 5417C centrifuge) at 4°C.

2. Remove supernatant and discard; keep soil pellet.

3. Add 1 mL 1X PBS (pH 8) and vortex lightly to wash soil (steps 3 & 4 optional)

4. Centrifuge 5 minutes at 2500xg at 4°C. Remove supernatant and discard.

5. Transfer the soil pellet (0.5g) to Lysing Matrix Tube E using a metal spatula that has been flame sterilized to remove RNases. Proceed to Step 6 (modified from Griffiths et al., 2000).

6. Working in fume hood, add 10μL β-mercaptoethanol, 0.5mL CTAB buffer and 0.5mL phenol-chloroform-isoamyl alcohol pH 8 (25:24:1)

7. Disrupt cells using Disrupter Genie for 2 minutes (in fume hood) break up total time in intervals of 30s by placing samples on ice for 30s.

8. Centrifuge 12000 rpm (15,000xg) for 5 minutes at 4°C (in fume hood).

9. Remove supernatant to clean 2mL tube, avoid volume at phase interface.

10. Add equal volume of chloroform-isoamyl alcohol (24:1), mix by gently inverting tube several times.

11. Centrifuge 12000 rpm (15,000xg) briefly to allow phase separation (in fume hood)

12. Transfer supernatant to a clean 2.0 mL tube.

13. Repeat chloroform-isoamyl extraction (steps 10-12)

14. Add 1μL glycogen (20mg/ml) and 1 volume 20% PEG 6000 in 2M NaCl (final 10% PEG-1M NaCl) (modified from Griffiths et al 2000).

15. Precipitate nucleic acids -20˚C for 2 hours.

16. Centrifuge 18,000xg (13,000 rpm) for 10 minutes at 4˚C (tip: orient tubes to reliably locate nucleic acid pellet, allowing care to be taken to minimize losses during steps 17 and 18)

17. Remove supernatant (tip: orient pellet “up” in tube while removing supernatant carefully with pipettor to avoid accidentally aspirating nucleic acid into liquid volume)

18. Wash pellet with 70% ice cold ethanol and air dry (do this carefully to avoid losing pellet) under UV prep hood.

19. Resuspend pellet in *50-200μL RNase/DNase-free water. Transfer suspension to fresh 0.5-ml microfuge tube. To facilitate solubilization of RNA, suspension can be incubated at 65°C, 5 min. This is your crude extract. Excessive care must be taken to avoid nucleases hereon out.

*Volume selected for resuspension of nucleic acids is based upon expected yield. Use a higher volume if a high nucleic acid yield is anticipated.

20. Quantification and quality assessment for DNA and RNA can be performed on crude extract using Qubit and agarose gel electrophoresis.

21. Following DNA quantitation, remove a portion of the crude extract as DNA fraction into a separate fresh 0.5-ml microfuge tube. Store in -20°C freezer until further use.

22. Proceed with DNase digestion to obtain purified RNA from crude extract. Remove desired volume of DNA/RNA fraction and proceed with DNase digestion as below. Storage of RNA for any length of time beyond quantifying is not recommended, but is possible at -80°C.

*S1.2. Fluidigm Access Array Protocol*

Prior to amplification, the genomic DNA was diluted to 2 ng/μL. Two rounds of PCR were performed to integrate both locus specific primers and Fluidigm/Illumina linkers and barcodes, respectively. All samples were run on a Fragment Analyzer (Advanced Analytics, Ames, IA) and amplicon regions and expected sizes confirmed. Samples were then pooled in equal amounts according to product concentration. The pooled products were then size selected on a 2% agarose E-gel (Life Technologies) and extracted from the isolated gel slice with Qiagen gel extraction kit (Qiagen). Cleaned size selected products were run on an Agilent Bioanalyzer to confirm appropriate profile and determination of average size. The DNA from the entire Fluidigm array was quantified and sequenced on one NovaSeq 6000 flowcell for 301 cycles from each end of the fragments using a NovaSeq 600-cycle sequencing kit version 3. Fastq files were generated and demultiplexed with the bcl2fastq v2.20 Conversion Software (Illumina). PhiX DNA was used as a spike-in control and removed in the data processing. Read lengths were 250 nucleotides. The raw data was sorted by the PCR-specific primers and paired end reads were obtained and demultiplexed by sample index.

*S1.3. Sequence Processing*

All sequences were analyzed using mothur [3] and then imported to Phyloseq [4]. Please refer to the tutorial on the mothur Wiki (https://mothur.org/wiki/MiSeq_SOP) to better understand the commands used here. We ran these analyses on High-Performance Biological Computing of UIUC, and typically used 8 processors and 96 GB of memory (RAM) for each run (300 GB of memory was used for the 16S rRNA genes due to the large reference database and the heterogeneity of the gene). We have included FASTA files of all the reference alignments used for the sequences of target genes listed in Table 1. Amplicon length, reference sequence file and aligned length are shown in Table S10 below. After running mothur, output files downloaded for subsequent statistical analysis and sequence alignment were: 1) the “.shared” file which contains the OTU abundance in each sample analyzed clustered at 97% sequence similarity, 2) the “.taxonomy” file which represents the taxonomic levels of obtained OTU sequences, 3) the “.rarefaction” file with alpha-diversity metrics, and 4) alpha-diversity metrics for normalized output (“ave_std.summary”) and non-normalized output (“groups.summary”).

*S1.4 Statistical comparisons of sequences from 2014 and 2019 samples*

Since the 16S rRNA amplicon sequences are generated in different sequencing platform (2014 samples: Miseq v2.0, 2019 samples: Novaseq) and used different primers (2014 samples: V4 for bacteria [5] and Arch349f [6] for archaea; 2019 samples: Arc519f-Bac785r [7] for archaea and bacteria), those obtained sequences were processes separately in mothur using the same batch file, which we align the sequences to the same reference sequence file (SILVA 138 database) and the same taxonomy file. To further correct for the batch effects, we compare the unique populations of 2014 and 2019 samples in taxonomic levels higher than genus level by “tax_glom” function in phyloseq. This dataset was used to determine the alpha and beta diversity as well as the relative abundance changes in 2014 and 2019 samples. To compare the effectiveness of different batch effect correction methods, we also subsample the 2019 samples to have similar sequencing depths as the 2014 samples. Using beta analysis to compare 2014 and 2019 samples, we found that both methods show similar disparities between 2014 and 2019 samples (Figure 2, Supplementary Figure S10), suggesting that we have corrected our samples with appropriate bias.

**S2. Supplemental results**

S2.1 Other physiochemical changes in 2014 to 2019

Other parameters that change significantly from 2014 to 2019 are likely linked to the change in salinity. We observe significant positive correlations between both alkalinity and K with salinity (alkalinity: *r* = 0.77, *p* < 0.01; K: *r* = 0.94, *p* < 0.01). Positive correlations are expected, since K partly comprises salinity, while alkalinity shows positive correlations with salinity in other lakes [8]. We also find that Ca has a significant negative relationship with salinity (*r* = -0.74, *p* < 0.01). Only one previous study reported a negative correlation between salinity and Ca [8]. As a result of constant weathering from the limestone bedrock on Kiritimati Island, the lacustrine system can have a high flux of calcium ions from groundwater, which lead to it deviating from other solutes. This flux of Ca would be diminished in times of reduced rainfall and groundwater recharge. Furthermore, abiotic and biotic carbonate precipitation and dissolution may also affect the Ca/salinity ratio [9, 10]. The δ^18^O values of the lake water shows an insignificant correlation with salinity (*r* = 0.03, *p* = 0.88). Since the thermodynamic activity of water decreases at higher salinity [11], the associated kinetic fractionation that increases δ^18^O values of the residual water will also be lower during this time, leading to lower lake water δ^18^O values and a weaker correlation between lake water salinity and δ^18^O values. The spatial survey of 2019 lakes supports this ‘salt effect’ when salinity exceeds 60 ppt (Figure S6). In conclusion, except for δ^18^O, it is likely that salinity can also explain many other physiochemical parameters that changed significantly from 2014 to 2019.

References:

1. Griffiths RI, Whiteley AS, O&apos;Donnell AG, Bailey MJ. Rapid method for coextraction of DNA and RNA from natural environments for analysis of ribosomal DNA- and rRNA-based microbial community composition. *Appl Environ Microbiol* 2000; **66**: 5488–5491.

2. Paulin MM, Nicolaisen MH, Jacobsen CS, Gimsing AL, Sørensen J, Bælum J. Improving Griffith’s protocol for co-extraction of microbial DNA and RNA in adsorptive soils. *Soil Biol Biochem* 2013; **63**: 37–49.

3. Schloss PD, Westcott SL, Ryabin T, Hall JR, Hartmann M, Hollister EB, et al. Introducing mothur: Open-source, platform-independent, community-supported software for describing and comparing microbial communities. *Appl Environ Microbiol* 2009.

4. McMurdie PJ, Holmes S. phyloseq: an R package for reproducible interactive analysis and graphics of microbiome census data. *PLoS One* 2013; **8**: e61217.

5. Caporaso JG, Lauber CL, Walters WA, Berg-Lyons D, Lozupone CA, Turnbaugh PJ, et al. Global patterns of 16S rRNA diversity at a depth of millions of sequences per sample. *Proc Natl Acad Sci U S A* 2011; **108**: 4516–4522.

6. Takai K, Horikoshi K. Rapid detection and quantification of members of the archaeal community by quantitative PCR using fluorogenic probes. *Appl Environ Microbiol* 2000; **66**: 5066.

7. Klindworth A, Pruesse E, Schweer T, Peplies J, Quast C, Horn M, et al. Evaluation of general 16S ribosomal RNA gene PCR primers for classical and next-generation sequencing-based diversity studies. *Nucleic Acids Res* 2013; **41**: e1–e1.

8. Kebede E, Mariam ZG, Ahlgren I. The Ethiopian Rift Valley lakes: chemical characteristics of a salinity-alkalinity series. *Hydrobiol 1994 2881* 1994; **288**: 1–12.

9. Dupraz C, Reid RP, Braissant O, Decho AW, Norman RS, Visscher PT. Processes of carbonate precipitation in modern microbial mats. *Earth-Science Rev* 2009; **96**: 141–162.

10. He H, Li Y, Wang S, Ma Q, Pan Y. A High Precision Method for Calcium Determination in Seawater Using Ion Chromatography. *Front Mar Sci* 2020.

11. Gonfiantini R. Environmental isotopes in lake studies. *Terr Environ B* 1986; 113–168.

**Supplemental Table Captions (Tables S1-S9 are in the excel file)**

**Table S1:** Primer sets used for 16S rRNA, N- and S-cycle functional genes forward and reverse sequences

**Table S2:** Modern water chemistry values from surface survey on Kiritimati, black color represents samples collected in both 2014 and 2019, and red color represents samples collected only in 2019

**Table S3:** Median (with ±1SD) values of physiochemical parameters measured in 2014 and 2019 samples, p values indicate results from pairwise wilcoxon test and adjusted p values are calculated from fdr

**Table S4**: Correlation coefficient of alpha diversity with environmental factors, bold p-values indicate significant p values < 0.05

**Table S5:** Summary of number of high-throughput sequencing reads recovered after quality trimminga. Functional genes denoted with “*” are samples that have been subsampled using sub.sample from mothur pipeline. “-” means that the detected reads were low, or they could not be classified as the targeted genes.

**Table S6:** Mantel test for 16S rRNA genes with environmental parameters. P values adjusted from fdr. Bold p-values indicate significant p values < 0.05.

**Table S7:** Comparisons of median (with ±1SD) values of relative abundances of metabolisms (indicated from 16S rRNA gene sequence analysis using FAPROTAX) in 2014 and 2019 samples. p values indicate results from pairwise wilcoxon test and adjusted p values are calculated from fdr corrections. bold p-values indicate significant p values < 0.05

**Table S8:** Mantel test results of metabolisms predicted from FAPROTAX with environmental factors. P values adjusted from fdr. bold p-values indicate significant p values < 0.05. P-values lower than 0.0001 are shown as <0.0001.

**Table S9:** Mantel test for functional gene results with microbial communities (16S rRNA gene results), p values adjusted by fdr. bold p-values indicate significant p values < 0.05

**Table S10:** Correlation coefficient of relative abundance of genera from Halobacterota phylum with salinity, bold p-values are genera that present in at least half (11) of the samples

**Table S11**: Amplicon length, reference sequence file and aligned length for targeted genes

List of NosZ gene reference files, amplicon length and alignment length used for the sequence analysis. This information can be plugged into the **Batch** file above. Reference files were uploaded as **fasta** files in the supplemental materials folder.

| Gene | Amplicon Length | Gene Reference File | Alignment Length |
| --- | --- | --- | --- |
| nifH | 362 | nifH-REF_aligned.fasta | 368 |
| nirK | 430 | NirKC1_REFs.fasta | 430 |
| nirS | 430 | NirSC1_REFS.fasta | 430 |
| nrfA | 240-270 | NrfAREFalign-Muscle.fasta | 419 |
| nxrB | 485 | nxrB-REF_aligned.fasta | 485 |
| nosZ | 254 | NosZREF-CladeI.fasta | 254 |
| amoA (AOA) | 292 | AOA-amoA-REF.fasta | 295 |
| amoA (AOB) | 489 | AOBamoA-REF_aligned.fasta | 489 |
| soxB | 362 | soxB-REF2.fasta | 362 |
| aprA | 395 | aprA-REF_aligned.fasta | 395 |

**Supplemental Figures**

**
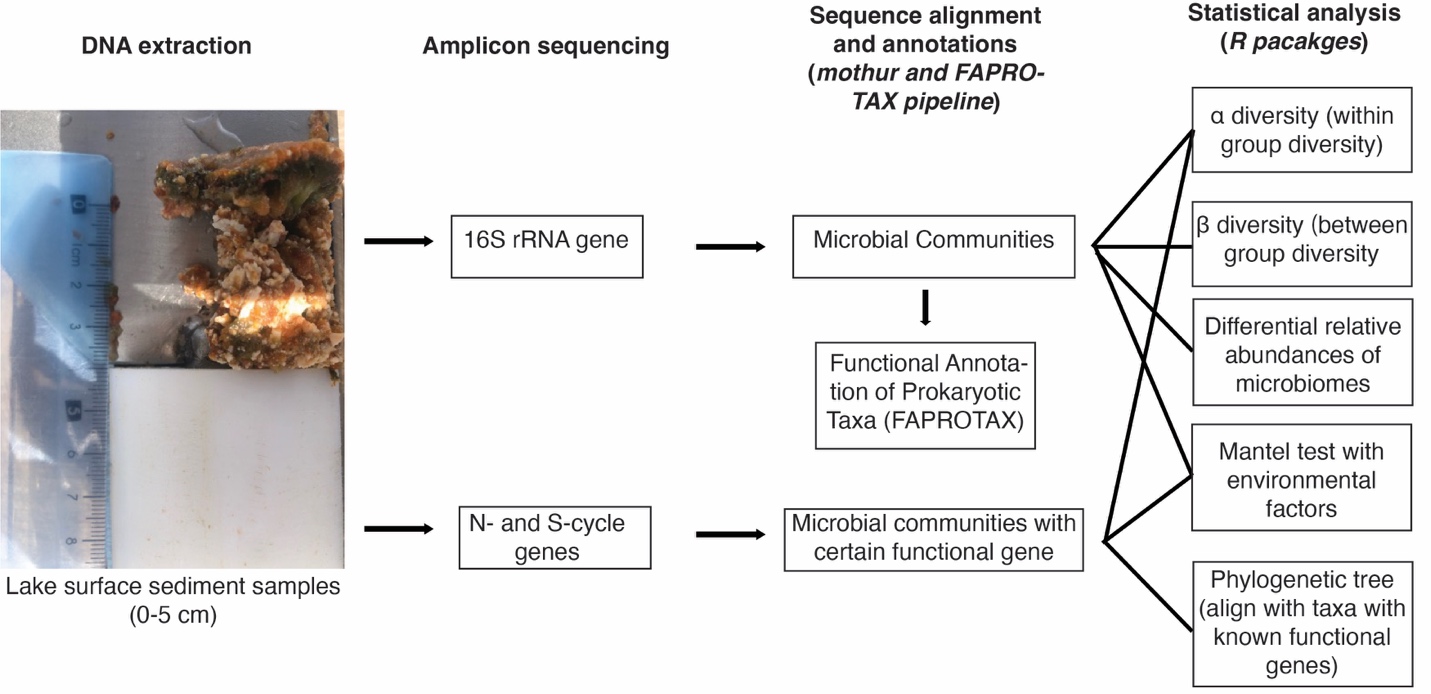
**

**Figure S1:** Conceptual diagram showing the major analysis used in this manuscript

**Figure S2**: Principal component analysis (PCA) of environmental factors measured in lake samples of 2014 and 2019.

**Figure S3**: Correlogram of all measured geochemical variables. The color bar indicates correlation coefficient from -1 to +1, and the size of the colored circle is proportional to the strength of correlation. Blank grids indicate p > 0.05.


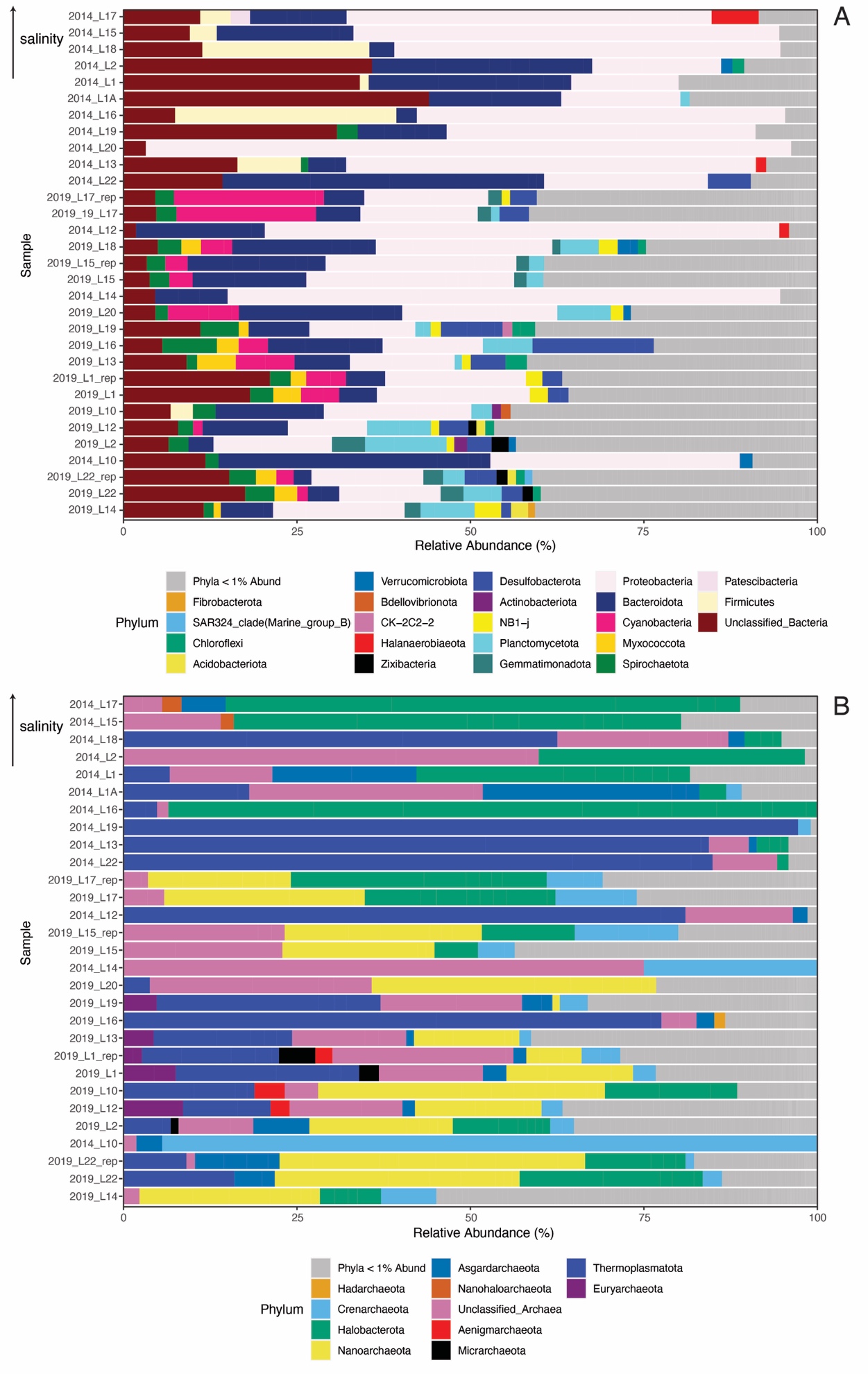


**Figure S4**: (A) Bacterial and (B) archaeal communities determined at phylum level based on high-throughput sequencing of the V4 hypervariable region of the 16S rRNA genes from 33 lake surface sediment samples collected from 2014 and 2019 field trips. In (A) and (B), “Phyla < 1% Abund” indicate minor phyla that consists of < 1% of total phyla identified in the samples.


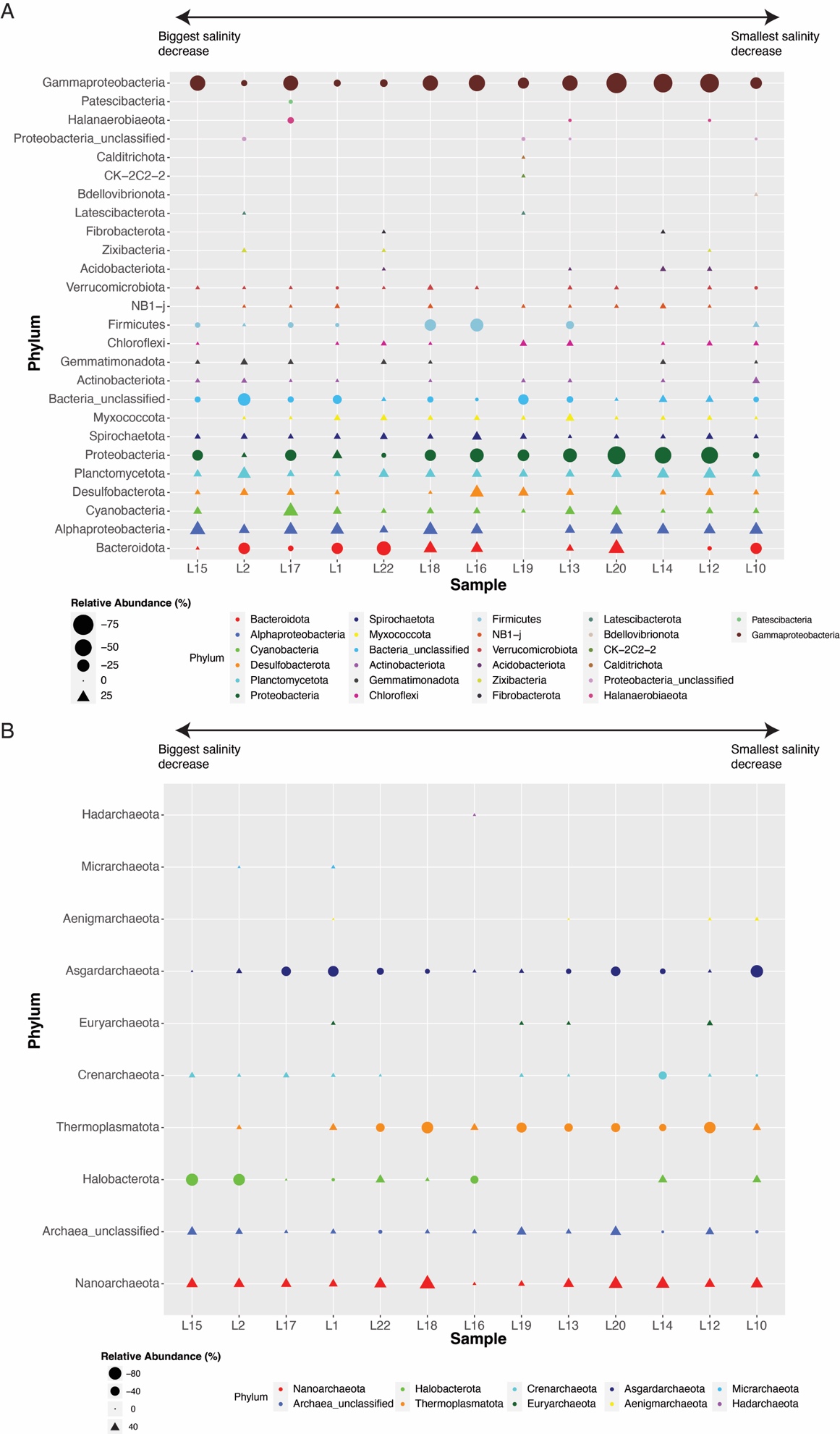


**Figure S5**: Lake-specific microbial community comparisons for 2014 and 2019 samples, showing differences of the relative abundance of the most abundant (A) bacterial and (B) archaeal phyla in Kiritimati sediment samples which are listed on x-axis. Circle symbols represent higher relative abundances in 2014 samples, and triangle symbols represent higher relative abundances in 2019 samples. Lake samples are placed in the x axis in the order of most decrease in salinity to least decrease in salinity.


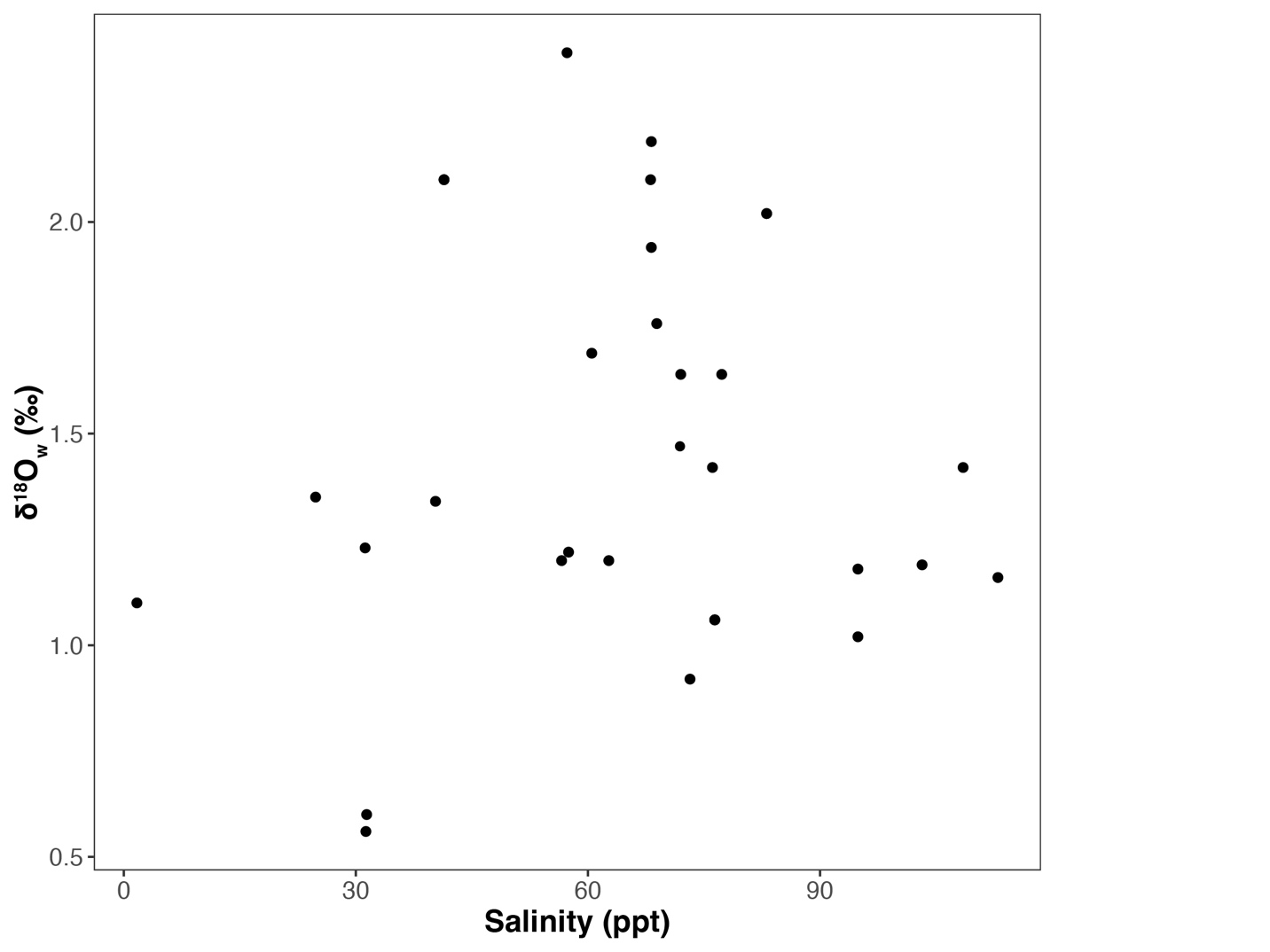


**Figure S6**: Scatter plot of δ^18^O_w_-salinity.

**Figure S7:** Correlogram of alpha diversity of different functional genes with measured environmental variables. The color bar indicates correlation coefficient from -1 to +1, and the size of the colored circle is proportional to the strength of correlation. Blank grids indicate p > 0.05.

**Figure S8**: Distribition of *aprA* OTUs in the Kiritimati sediments under investigation and the phylogenetic tree for the *aprA* genes. The phylotypes under consideration (blue) comprised of the top 5 abundant OTUs identified in all the samples. The relative abundances of OTUs are presenting using a log2 transformation. The reference *aprA* genes (pink) were identified via BLAST against the NCBI-non redundant database and the gene sequences for the cultivated species were preferentially selected.

**Figure S9:** Distribition of *nifH* OTUs/sequences in the Kiritimati Lake 1 sediments under investigation and the phylogenetic tree for the *nifH* genes. The phylotypes under consideration comprised of the top 5 abundant OTUs identified in Lake 1 in 2019 (red), annotated *nifH* genes of assembled metagenomes from MET-1 of IMG (black), and annotated *nifH* genes from metagenom-assembled genomes from MET-1 (pink). The reference *nifH* genes (blue) were identified via BLAST against the NCBI-non redundant database and the gene sequences for the cultivated species were preferentially selected.

**
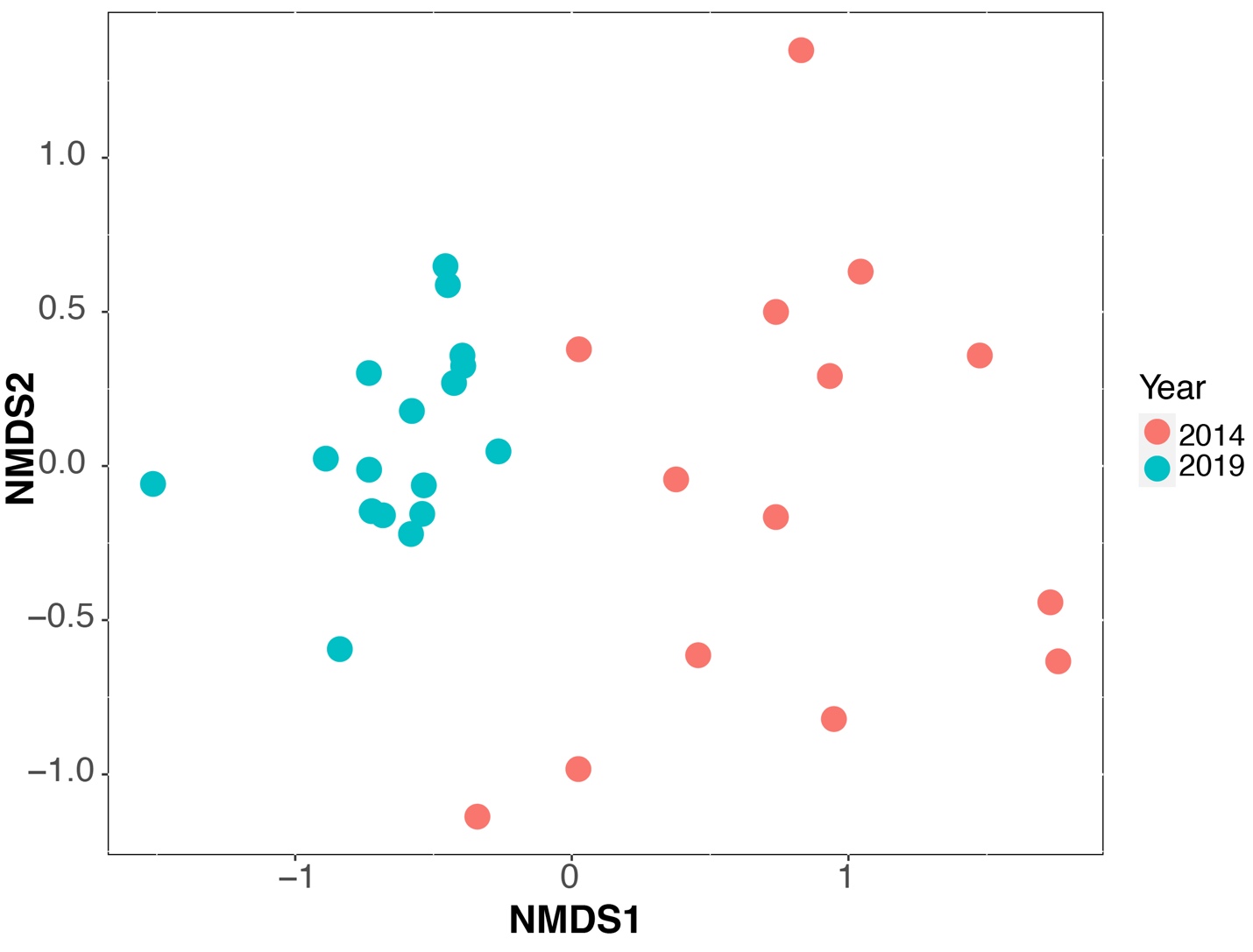
**

**Figure S10:** Non-metric multidimensional scaling (NMDS) plots of bacterial based on 16S rRNA gene sequencing data, using another method to correct for batch effects.
